# A typical case of hydrallantois accompanied by fetal monstrosity in a local ewe of Kashmir

**Published:** 2012

**Authors:** Hiranya Kumar Bhattacharyya, Shahid Hussain Dar, Mujeeb-ur-Rehman Fazili, Abdul Hafiz

**Affiliations:** *Faculty of Veterinary Sciences and Animal Husbandry, Sher-e-Kashmir University of Agricultural Sciences and Technology, Shuhama-Alusteng, Srinagar -190 006, Jammu and Kashmir, India.*

**Keywords:** Hydrallantois, Fetal monstrosity, Ewe

## Abstract

A full termed local ewe with the history of continuous straining with labored breathing for last 24 hours was presented. The animal was disinclined to move with tense and round abdomen which developed rapidly during last two weeks. Caesarean section revealed hydrallantois accompanied by multiple fetal congenital abnormalities. The ewe was under observation for four weeks. Metritis developed 12 days post-operation and was treated successfully. The ewe was found active on 25 days post-surgery with gain of extra 3 kg bodyweight.

## Introduction

Hydrallantois is frequently reported in bovines. It is rarely reported in small ruminants. In such a condition, the fetus is either normal or slightly smaller in occasional cases.^1^ No report on fetal monstrosity along with hydrallantois is available in the literature. The present report documents a rare case of hydrallantois along with fetal monstrosity in a local sheep of Kashmir. 

**Case history observation and treatment**

A full termed local ewe, aged 3 years and weighing 48 kg, at her first parity was presented with the history of labor started 24 hours earlier. As per history there was rapid abdominal enlargement in last 15-20 days which was suspected as a probable cause of twin pregnancy by the owner. At the time of presentation the animal was in sternal recumbency. Abdominal wall was bilaterally distended, round and tense. Pulse rate was elevated (100 bpm) with normal temperature (38.6 ˚C) and deep abdominal respiration. Vaginal examination revealed closed os-cervix. Ultrasonography revealed heavy fluid accumulation in the allantoic cavity. The case was initially diagnosed to be a case of hydrallantois. Because of the condition of the patient it was decided to perform immediate cesarean section. Pre-operatively animal was administered with analgesic, systemic antibiotic and intravenous fluid therapy as per recommended therapeutic dose. 

**Fig. 1:**
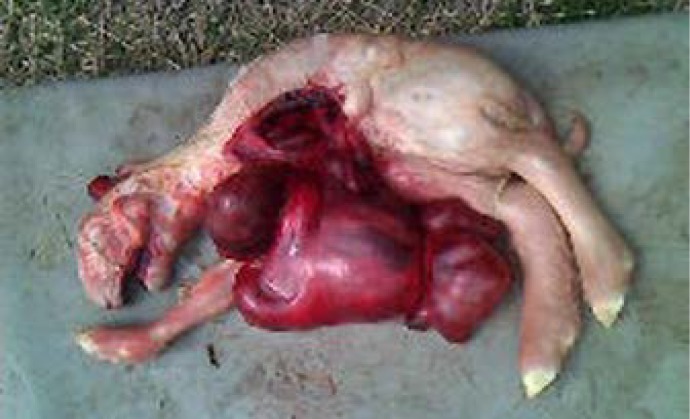
Monster with multiple congenital defects

The C-section was conducted under local infiltration of 2% lignocaine hydrochloride using left paramedian approach as per standard procedure. Fluid filled gravid uterus was very cautiously exteriorized with utmost care to prevent peritoneal contamination. After incising the uterine horn, more than 15 liters of amber colored allantoic fluid resembling transudate were drained out. Following removing of fluids a male monster (Fig. 1) was taken out. Placenta was separated from the caruncles cautiously. Some of the placentae were enlarged. The horns were lavaged with mild antiseptic solution and two clenex boli (1 blous contains 60mg nitrofurazone, 100 mg metronidazole, 6 g urea and 60 mg povidine iodine, Animal Health, Dosch Pharmaceuticals Pvt. Ltd., Mumbai, India) were inserted intrauterine. The uterus was sutured with chromic catgut No. 1 using Cushing pattern. Muscle and skin were sutured routinely. Body weight of the dam following surgery was found only 25 kg. Post-operatively, intramuscular Fetitas 1 mL (each mL contains 50 mg iron sorbitol citric acid, 500 µg folic acid and 50 µg cyanocobalamin, Intas Pharmaceuticals Ltd., Ahmedabad, India), systemic antibiotic(amoxicillin+cloxacillin 250 mg IM bid for 7 days), analgesic (meloxicam 0.2 mg kg^-1^, IM for 3 days) were administered with regular antiseptic dressing. Information of the patient was taken by phone at every two days interval. Skin sutures were removed 12 days following surgery. However, metritis was developed and was treated with both systemic and intrauterine antibiotic preparations with a single dose of PGF_2_α. The animal was found active and alert 25 days post-operation with 3 kg extra body weight gain. 

**Fig. 2:**
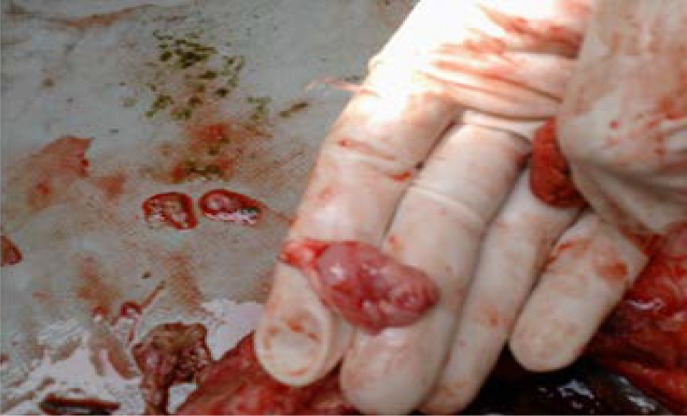
Underdeveloped brain of the monster

**Description of the monster. **The monster weighing 2.5 kg was alive up to 2-3 min and died thereafter. Before death heart beat could be heard very prominently as part of it was extrathoracic. The head of the monster was connected with blood vessels and tissues to umbilicus. Whole intestines and liver were herniated through a big opening at the site of attachment of the absent left (Amelia) fore leg to the body. There was cleft palate and both the mandibles were separated up to canthus. Left sided ribs were also absent (Fig. 1). On sectioning brain underdeveloped devoiding of two distinct hemisphere and prominent sulci and gyri were not found (Fig. 2). 

## Discussion

Hydrallantois is excessive accumulation of fluid in the allantoic sac. It may result from abnormal functioning of the placentomes due to inadequate numbers of caruncles and development of adventitial placentation.^2^ In cattle it may also be due to infection caused by bovine viral diarrhea virus or BVDV.^3^ Hydrallantois also occurs sporadically in dairy and beef cattle; its occurrence is very rare in small ruminants. It has been reported in cattle carrying twin fetuses and is associated with diseased uterus in which caruncles are not functional and rests of the placentomes are enlarged and diseased. However, in these cases fetuses are usually slightly smaller than normal and show some edema and ascities.^1^ Enlargement of some of the placentomes were also noticed in the present case. Hydrallantois with fetal defects is not reported in the available literature. Instead normal fetus was reported in these findings.^1^^,^^4^

Various methods to induce parturition in cattle suffering from hydrallantois is reported in the literature including use of cloprostenol or other PGF_2_α preparation and estrogen preparations.^5^^,^^6^ However, C-section is best choice when it is felt that the animal may not withstand the stress imposed by normal delivery provided that prognosis of the animals is not grave. When prognosis of the animal is grave caesarean is not recommended but may be tried to save the life of the valuable animals. Delivery of two lambs by caesarean from a ewe suffering from hydrallantois is also reported.^6^

Hydrallantois can easily be diagnosed by rectal palpation in bovine with the findings of distorted uterine horn and no palpation/ballottement of fetus and placentomes but this is not possible in small ruminants.^1 ^Ultrasonography can be a very useful alternative to diagnose the condition. Moreover, rapid abdominal enlargement in last 15-20 days with round distended and tense abdominal wall are characteristic features of hydrallantois which was also noticed in the present case. Acute, progressive, bilateral abdominal distention was also observed in a ewe suffering from hydrallantois.^6^ In the present case it was confirmed on exploratory surgery with the presence of amber colored huge amount of allantoic fluid. Occurrence of metritis following removal of fetus and allantoic fluid was also reported earlier in cattle.^1^ Previous report indicated nonoccurrence of retained fetal membrane and septic metritis in ewe suffering from hydrallantois.^6^

Characteristic findings of the monster recorded in this study were somewhat similar to that of schistosomus reflexus. However, classical schistosomus reflexus is characterized by marked ventral curvature of spine, lateral bending of fetal body and chest wall exposing thoracic and abdominal viscera with deformed pelvis, ankylosis of limbs and enclosure of head in a complete sac of skin.^7^ In routine handling of dystocia cases, schistosomus reflexus can easily be diagnosed by either feeling the viscera, which might be mistaken for dam’s viscera by the operator’s hand or even a beating heart. This misjudgment can be avoided by careful examination of the fetus. 

## References

[1] Robetrs SJ Veterinary Obstetrics and Genital Diseases (Theriogenology).

[2] Maxie MG ( 2007). Female genital system. Pathology of Domestic Animals.

[3] Collyer JH (1990). Hydrallantois in cows. Vet Rec.

[4] Noakes ED, Parkinson TJ, England GCW ( 2001). Arthur’s Veterinary Reproduction and Obstetrics.

[5] Sharp AJ, Bierschwal CJ, Elmore RG (1978). A case report response of two cows with hydramnios and hydrallantois to treatment with cloprostenol. Theriogenology.

[6] Peiro JR, Borges AS, Yanaka R (2007). Hydrallantois in an ewe (case report). ARS Veterinaria, Jaboticabal.

[7] Wani NA, Wani GM, Bhat AS (1994). Schistosomus reflexus in a Corriedale ewe. Small Rumin Res.

